# Spectral induced polarization (SIP) measurements across a PFAS-contaminated source zone

**DOI:** 10.1016/j.jhazmat.2024.135829

**Published:** 2024-09-15

**Authors:** Ethan Siegenthaler, Sam Falzone, Charles Schaefer, Dale Werkema, Lee Slater

**Affiliations:** a Department of Earth and Environmental Sciences, Rutgers University Newark, NJ, USA; b CDMSmith, Edison, NJ, USA; c Environmental Protection Agency (EPA), Newport, OR, USA; d Pacific Northwest National Laboratory, 902 Battelle Blvd., Richland, WA 99354, USA

**Keywords:** Aqueous film forming foam (AFFF), Poly- and per-fluoroalkyl substances (PFAS), Spectral induced polarization (SIP), Source zone characterization

## Abstract

There is a need to develop field-scale, in situ screening technologies for assessing variations in aqueous film-forming foam (AFFF) concentrations in soils at former fire training and storage sites. Field-scale Spectral Induced Polarization (SIP) geophysical measurements were acquired on a transect crossing an AFFF source zone. Soil samples were acquired to determine variations in poly- and per-fluoroalkyl substances (PFAS) concentrations in soils, characterize soil texture, and create triplicate soil columns for laboratory SIP measurements. Field and laboratory observations show that SIP measurements are sensitive to the concentration of AFFF constituents associated with soil pore surface area. The specific polarizability and the phase of the SIP measurements for the laboratory samples were linearly correlated with total soil-sorbed PFAS concentration. The phase from the field SIP measurements was highest over the location of maximum PFAS concentration measured on the laboratory samples. However, a significant correlation between field-measured phase and laboratory-measured total PFAS concentration still needs to be established. These observations, along with the demonstrated sensitivity of the SIP response to the removal of soil PFAS using a methanol wash procedure, support the case for SIP characterization of AFFF source zones.

## Graphical Abstract

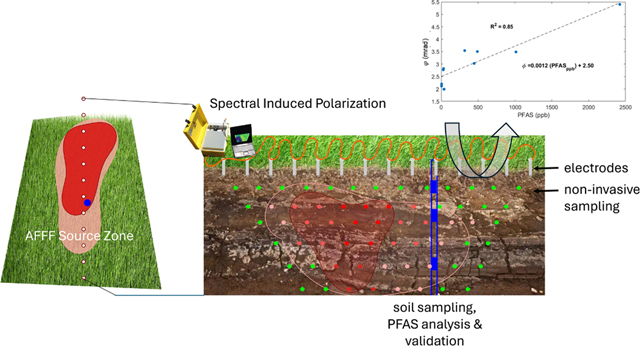

## 1. Introduction

The presence and nature of aqueous film-forming foam (AFFF), a widely used firefighting agent in soils, present a significant threat to groundwater [1,2]. Aqueous film-forming foam contains per- and polyfluoroalkyl substances (PFAS), known as hazardous substances. Sorption of PFAS onto soil particles plays a vital role in determining the fate and transport of PFAS substances, particularly concerning the cationic and zwitterionic forms [3–7]. These PFAS forms strongly correlate with soil phases, possibly becoming long-term contamination sources for groundwater through slow desorption and/or transformation.

Spectral Induced Polarization (SIP) is a non-invasive electrical geophysical technology that can, in some instances, be used to detect electrochemical alterations to the mineral-fluid interface that cannot be detected with conventional geophysical methods (e.g., the well-established electrical resistivity method). In this respect, the technique is foremost sensitive to sorption processes onto soils [8]. The deployment of this technique is non-invasive and very similar to the more commonly used electrical resistivity geophysical survey, whereby a pair of electrodes is used to inject electric current into the ground, and the resulting electric field gradient is measured between two pairs. Numerous previous studies have demonstrated the ability of SIP to detect the sorption of metals, nutrients, and other ionic contaminates to soils [9–11,8]. We hypothesize that SIP might be sufficiently sensitive to the sorption of PFAS and/or the persistent non-fluorinated hydrocarbon surfactants (HCS) associated with historically contaminated AFFF-impacted source areas [12]. The possible dependence on HCS, in addition to PFAS, encourages investigations of SIP as a screening technology for the characterization of AFFF-impacted areas. Preliminary investigations of the SIP response to artificial soils contaminated with AFFF and a small number (n = 3) of soil samples obtained from an AFFF source zone encourage further exploration of the technology [13,14]. While SIP (or any other geophysical technique) clearly cannot quantify PFAS concentration levels within source zones, the method might be applied to provide invaluable information on areas that may be more heavily impacted than others. This would reduce the number of samples needed and improve the chances of better quantifying PFAS soil source loads relative to blind sampling. This paper explores the possible use of SIP as a rapidly deployable, non-invasive geophysical technology to evaluate relative variations in soil-sorbed PFAS concentrations over an AFFF source zone.

## 2. The spectral induced polarization (SIP) method

**Fig. 1.**
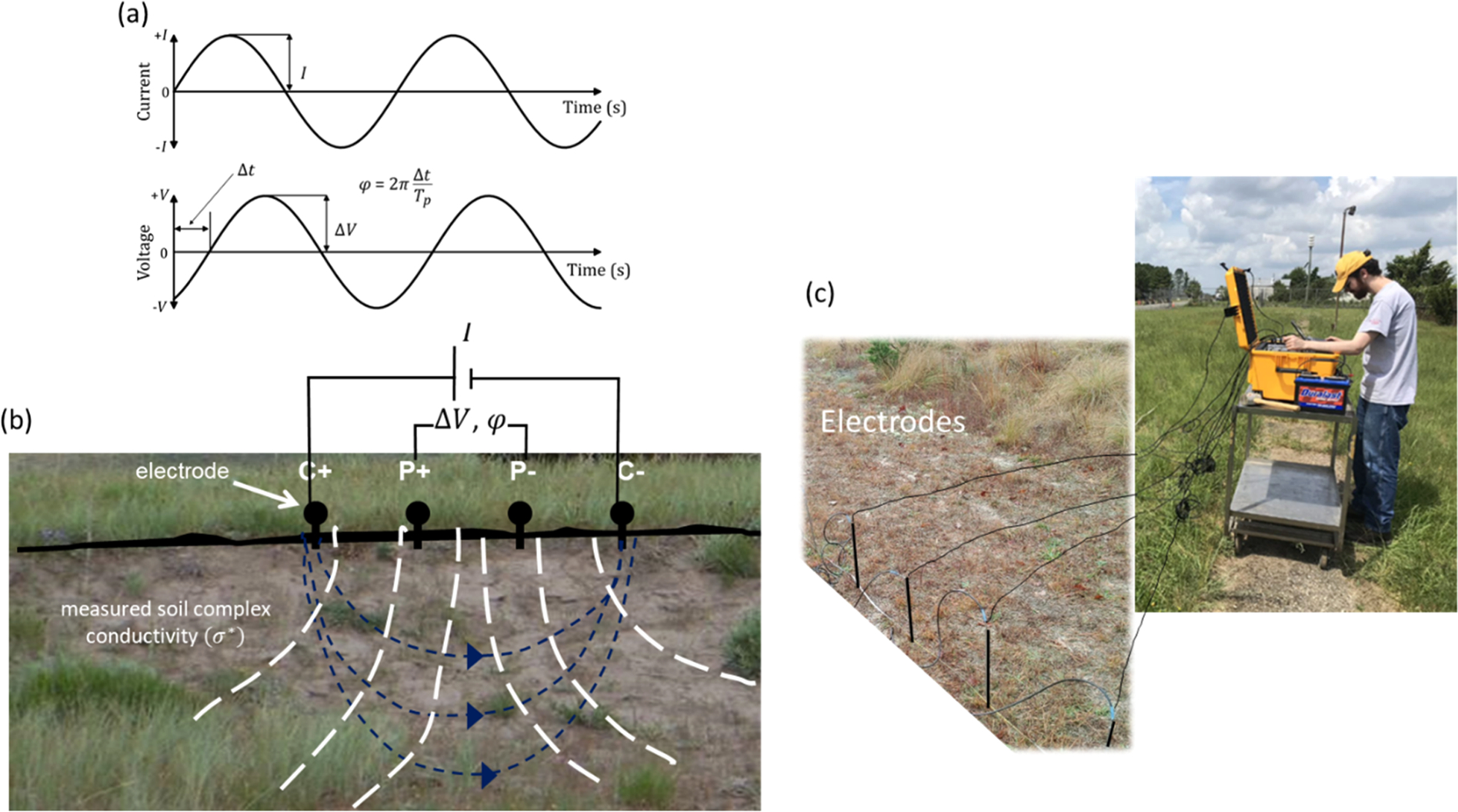
Concept of field SIP measurements (a) The voltage (ΔV) and phase lag (φ) across a pair of measuring electrodes is recorded relative to the current driven between a pair of injection electrodes (b) cartoon showing configuration of current injection pair (C+, C−) and potential recording pair (P +, P−) for field deployment (blue dashed lines represent idealized current flow paths, white hashed lines represent idealized equipotential lines) (c) photo of field deployment at an AFFF source zone site. In (a) $T_{p}$ represents the period of the applied waveform and Δt represents the time delay of the voltage waveform relative to the current waveform.

The SIP method involves measuring the frequency-dependent voltage magnitude (V) and phase lag (φ) between a pair of electrodes inserted into the Earth (or a column in the laboratory) relative to the electric current injected into the Earth (or a column) between a second pair of electrodes. These measurements are made over various frequencies to determine the Earth’s electrical conduction and polarization characteristics (Fig. 1).

The SIP measurements can be converted to a measured complex conductivity (σ*) via a geometric factor determined by the relative locations of the four electrodes on the Earth (or in a laboratory column). The complex conductivity can be described by the conductivity magnitude |σ|, φ, real ($\sigma^{\prime }$), and imaginary ($\sigma^{\prime \prime }$) parts of the complex conductivity. The real part of the complex conductivity represents the electromigration of charge carriers, whereas the imaginary part represents the temporary, reversible storage of electric charge. The phase is approximately the ratio of the polarization to the conduction strength,

(1) $$\varphi ={tan}^{-1}\left(\frac{\sigma^{\prime \prime }}{\sigma^{\prime }}\right)\approx \frac{\sigma^{\prime \prime }}{\sigma^{\prime }},$$

where the approximation is reasonable for φ<0.1 radians. At the low frequencies (< 1000 Hz) used for SIP measurements and in the absence of electron-conducting minerals, charge transport is primarily via electromigration and typically φ<0.03 radians.

Electromigration (or conduction) of charge in typical soils devoid of significant electron-conducting minerals occurs via two ionic mechanisms: (1) electrolytic conduction ($\sigma_{el}$) through the fluids occupying the interconnected pore network; and (2) frequency-dependent surface conduction ($\sigma_{\mathrm{surf}}^{*}$) within the electrical double layer (EDL) forming at mineral-fluid interfaces. Polarization (charge storage) below 1000 Hz is attributed to temporary and reversible charge displacement in the EDL. Assuming that electrolytic and surface conduction mechanisms are added in parallel,

(2) $$\sigma^{*}=\sigma_{el}+\sigma_{\mathrm{surf}}^{*},$$

(3) $$\sigma^{\prime }=\sigma_{el}+\sigma_{\mathrm{surf}}^{\prime },$$

and,

(4) $$\sigma^{\prime \prime }=\sigma_{\mathrm{surf}}^{\prime \prime }.$$

The electrolytic conductivity is the product of the pore-filling fluid conductivity and the electrical formation factor (F), which quantifies the reduction in the conductivity of the porous medium relative to the porefilling fluid and is controlled by the porosity and the pore connectivity [15,16]. The frequency-dependent surface conductivity is proportional to the surface-area-to-pore-volume ratio ($S_{por}$) divided by the formation factor [17]. As $\sigma^{\prime \prime }=\sigma_{\mathrm{surf}}^{\prime \prime }$,

(5) $$\sigma^{\prime \prime }-c_{p}\frac{S_{por}}{F},$$

where $c_{p}$ is defined as the specific polarizability [18,19]. The term $S_{por}/F$ describes the dependence of the surface conductivity on the physical properties of a soil while $c_{p}$ represents the influence of the electrochemistry, including surface charge density and mobility) on the EDL. Sorption influences $c_{p}$, which is potentially detectable with SIP measurements, foremost through $\sigma^{\prime \prime }$. Such sorption effects are not expected to be detectable with the established electrical resistivity method, which only senses the magnitude of the complex conductivity. Recognizing that $S_{por}$ is related to grain diameter ($d_{0}$) for a porous medium with porosity ∅ via,

(6) $$S_{\mathrm{por}}=\frac{1\;-\;\emptyset }{\emptyset }\frac{6}{d_{0}},$$

and assuming $\emptyset \approx F^{-1}$ (reasonable only for porous, unconsolidated, relatively well-sorted sediments), then,

(7) $$\sigma^{\prime \prime }\approx c_{p}\frac{F\;-\;1}{F}\frac{6}{d_{50}},$$

where the grain size for which 50% of the particle is finer ($d_{50}$) is taken to represent the mean particle size. Although not precise, Eq. 7 is more convenient to implement than Eq. 5 as the grain size of a soil sample is easily determined from particle size analysis, whereas measurements of $S_{por}$ require time-consuming/expensive measurements of specific surface area via gas adsorption apparatus.

The frequency dependence of $\sigma_{\mathrm{surf}}^{*}$ provides information on the length scales controlling the temporary storage of charge in a soil. Polarization of charge over smaller length scales is observed at higher frequencies, whereas polarization over larger length scales is observed at lower frequencies. The length scales controlling polarization of charge are related to the grain size and pore size distributions of a soil [20–22]. Consequently, the shape of the frequency spectrum can provide insights into grain or pore size distributions.

## 3. Site description

**Fig. 2.**
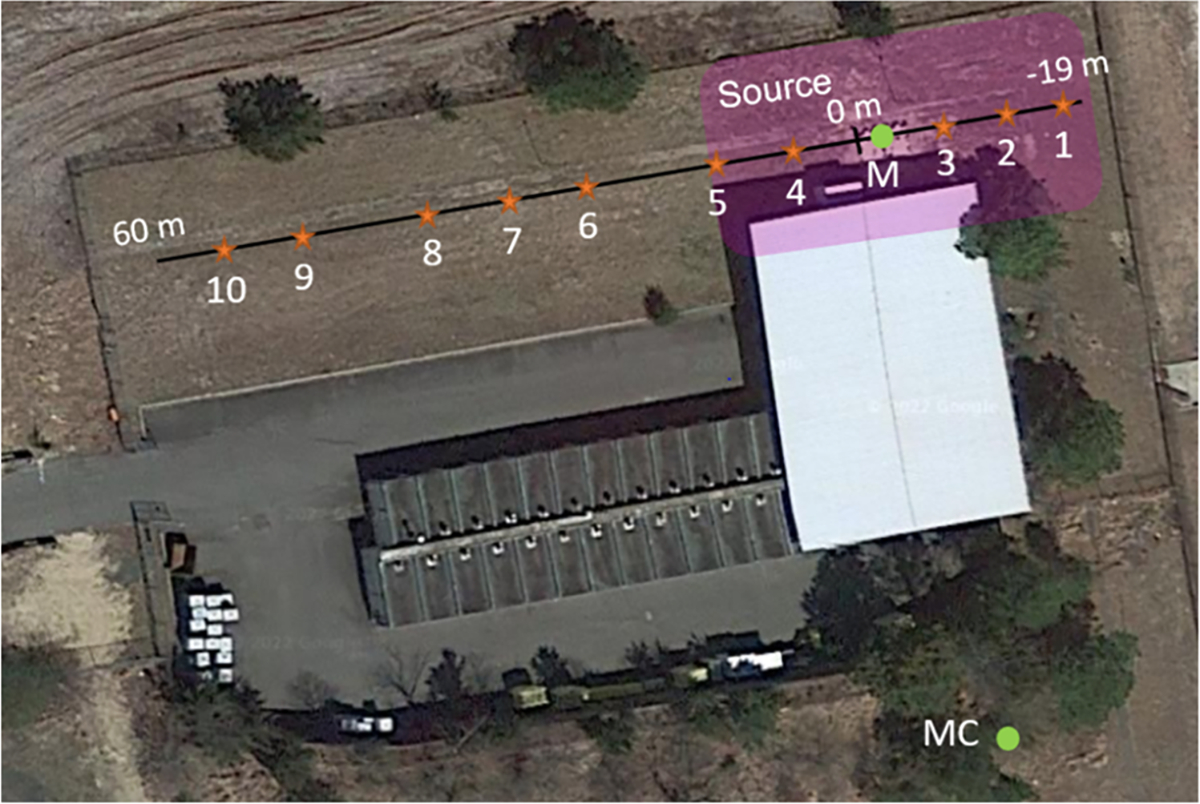
Aerial photograph of the PFAS source zone at the study site. The SIP survey line is shown in black, and soil sampling locations are shown with orange stars. Samples 1 – 10 and sample M were taken from within the vicinity of a known AFFF-impacted site. Sample MC was taken away from the source zone as a control.

This study was performed at a site historically impacted by AFFF releases, with reports of foam observed during heavy rainfall events. No firefighting activities have been documented in this area. Fig. 2 shows the hazardous waste storage and the possible extent of the AFFF source zone. A SIP transect across the site, along with the locations of 12 soil samples (labeled 1–10, M and MC), both described later, are also shown.

## 4. Methods

### 4.1. Field SIP survey

The SIP surveying was conducted at 3 m increments along the 79 m long transect crossing the source zone (Fig. 2). Measurements were acquired with a five-channel portable SIP instrument (Ontash & Ermac, NJ) (Fig. 1c). At each survey location, an array of eight electrodes spaced 0.3 m apart was established. The SIP measurements were collected using the dipole-dipole array, where a pair of electrodes at one end of the line is used for current injection (C+, C−) while subsequent electrode pairs (P +, P−) measure the voltage. As the distance between the current pair and the voltage recording pair (P +, P−) increases, the investigation depth of the measurements also increases, although the spatial resolution correspondingly decreases. Using approximate ‘depth of investigation’ rules based on a homogenous subsurface, e.g., that the investigation depth is equivalent to 0.195 times the length of distance between the outermost current electrode and outermost potential electrode of a dipole-dipole array [23], the investigation depth approximately varied from 0.18–0.40 m. This depth portion of the soil profile is approximately where AFFF contamination is located as determined from prior investigations and site history. More sophisticated, numerical methods are available to assess investigation depth and resolution better but are beyond the need of this study.

The SIP measurements at each location were simultaneously recorded for all five investigation depths over the frequency range 0.1 to 1000 Hz. This range was selected as (1) previous laboratory measurements on soil samples from the site [13,14] indicated strong sensitivity to PFAS sorption; (2) lower frequency measurements would be excessively time-consuming; and (3) higher frequency measurements would be excessively contaminated by instrumentation noise. The selected settings resulted in a data acquisition time of 11 min at each point along the profile.

### 4.2. Soil sampling

Soil sampling was performed along the transect that crosses the suspected extent of the source zone and beyond (Fig. 2). Soils were partially saturated at the time of sampling, with an average moisture content of 9.4% and a standard deviation of 1.63%. Sampling locations were selected to investigate variations in the SIP measurements recorded along the transect. Areas exhibiting high polarization (high $\sigma^{\prime \prime }$ and/or high φ) were targeted as these locations were hypothesized to be associated with high PFAS and/or non-fluorinated HCS concentrations in the soil. Areas of intermediate and low polarization response along the transect were also selected for comparison. A final sample was taken away from the source zone to provide an uncontaminated (or low contamination) control. An Environmental Protection Agency (EPA) approved Quality Assurance Project Plan (QAPP) was followed to facilitate PFAS sampling and analysis.

A decontamination site was set up 25 m away from the sampling location. This area was equipped with buckets for washing equipment with deionized water and Alconox/Liquinox. Equipment for sampling included a metal hand auger and small metal trowel, with HDPE sample containers. After each soil sample was acquired, equipment was brushed with deionized water and Alconox/Liquinox, and then with a final deionized water rinse to remove soils and chemical contaminants after every use to avoid any cross contamination between samples.

Soil samples were first acquired from suspected least contaminated locations (low polarization response), with suspected most contaminated locations (high polarization response) sampled later. A hand auger was used to collect samples of about 100 g each from the surface to a depth of about 12 cm. Individual samples were homogenized in a stainless-steel bowl with a metal trowel before being placed into labeled HDPE containers, which were placed in zip-lock bags. Sample bags were then placed in separate coolers on ice, with specific coolers designated for suspected higher contamination and lower concentration samples. The samples were stored in a cold room at 6 °C until they were shipped to a U.S. Department of Defense accredited laboratory, SGS AXYS Analytical Services Ltd. (Toronto, CA), for soil PFAS analysis. Method SGS AXYS MLA-110, released by the U.S. Environmental Protection Agency (EPA) as EPA Method 1633, was followed. In this method, soil samples are spiked with isotopically labeled surrogate standards, extracted in methanol, and cleaned up by carbon and SPE cartridges before analysis by liquid chromatography with tandem mass spectrometry (LC-MS/MS), with final sample concentrations determined by isotope dilution/internal standard quantification. The total soil PFAS concentrations reported in this work represent the sum of the target analytes used for this method, as reported in Table S1. The variation in soil texture along the transect was investigated by measuring the particle size distribution of each sample. Representative particle diameters were calculated based on the percentage mass of material finer than a particle size (e.g., d50 being the size for which 50% of the total soil mass is finer). The soil uniformity coefficient, Cu = d60/d10, representing the degree of sorting (small Cu values represent well-sorted soils), was also calculated.

### 4.3. Laboratory SIP measurements

Laboratory SIP measurements on the ten samples acquired from the field site were performed in triplicate to promote statistically significant results that account for differences due to variable packing in SIP experimental columns. Each field sample was thoroughly saturated with a synthetic groundwater representative of the study site (Table S2) to avoid differences in saturating fluids between samples. Given that the (predominantly silica) mineral surface will be negatively charged above pH 3 (the point of zero charge), we focused on matching the cationic composition known from inductively coupled plasma (ICP) spectroscopy analysis of a groundwater sample. We also matched the recorded specific conductance (80 *μ*S/cm) of the groundwater. The anionic concentration was not matched, as this would have required buffering.

The saturated sample material was then packed into three identical acrylic SIP columns (10 cm long, 2. 4 cm diameter) at 1 tsp (approximately 5 mL) increments, with material packed down between increments with a tamping tool. This procedure was repeated until all three SIP sample holders representing the soil sampled from one location on the transect were filled. This incremental wet packing procedure resulted in triplicate loosely packed columns with porosities ranging from 0.35–0.45 (mean of 0.41 and standard deviation of 0.03). The SIP measurements were made over an extended (relative to the field measurements) frequency range from 0.01 to 10,000 Hz. Experimental columns contained two coiled silver (Ag) electrodes placed at either end and point potential electrodes spaced 2.3 cm apart, with the midpoint between these electrodes located at the center of the columns.

### 4.4. Methanol wash procedure

A methanol wash procedure was conducted on samples from the site to investigate how the presence of PFAS influences the SIP response. The approach involved comparing the SIP response of a PFAS-contaminated sample from the AFFF source zone (total PFAS = 860 ppb) versus the same sample after a methanol wash procedure designed to remove PFAS from the soil (total PFAS reduced to 494 ppb). The same procedure was also performed on a soil sample remote from the source zone to serve as a control (total PFAS = 5.8 ppb before the wash; PFAS not detected after the wash). The two sample locations are shown in Fig. 2, with sample M being the contaminated sample and MC being the control. Batch soil washing was performed by shaking (95 RPM) 900 g of each soil with 2.3 L of methanol in a 100 L glass container for three days. After the initial three days of shaking, the supernatant methanol was removed, replaced with 2.3 L of fresh methanol, and shaken for an additional two days; this methanol replacement and shaking was repeated one time, bringing the total soil washing time to 7 days. After the final methanol washing phase, the supernatant methanol was removed, and the remaining bulk methanol was evaporated from the soil by drying in a fume hood overnight. The PFAS analysis using EPA Method 1633 was performed on unwashed and washed soils by SGS AXYS Analytical Services Ltd (Toronto, CA).

## 5. Results

### 5.1. PFAS concentrations along the transect

Total soil PFAS concentrations recorded at spot locations along the transect confirm the presence of high PFAS concentrations coinciding with the expected source zone based on historical data for the site. Total soil PFAS concentrations along the transect varied between 3 and 2421 ppb. The plot of PFAS variation along the transect is strongly influenced by the anomalously high value of 2421 ppb recorded at − 10 m along the line (Fig. 2), although total soil PFAS concentrations are overall orders of magnitude higher within the suspected source zone between −19 m and + 20 m (between 318–2421 ppb) relative to other locations (between 3.4–38 ppb). Of the 18–20 PFAS compounds detected in each sample, PFOS had by far the highest concentrations.

### 5.2. Single frequency SIP data versus PFAS soil concentrations

**Fig. 3.**
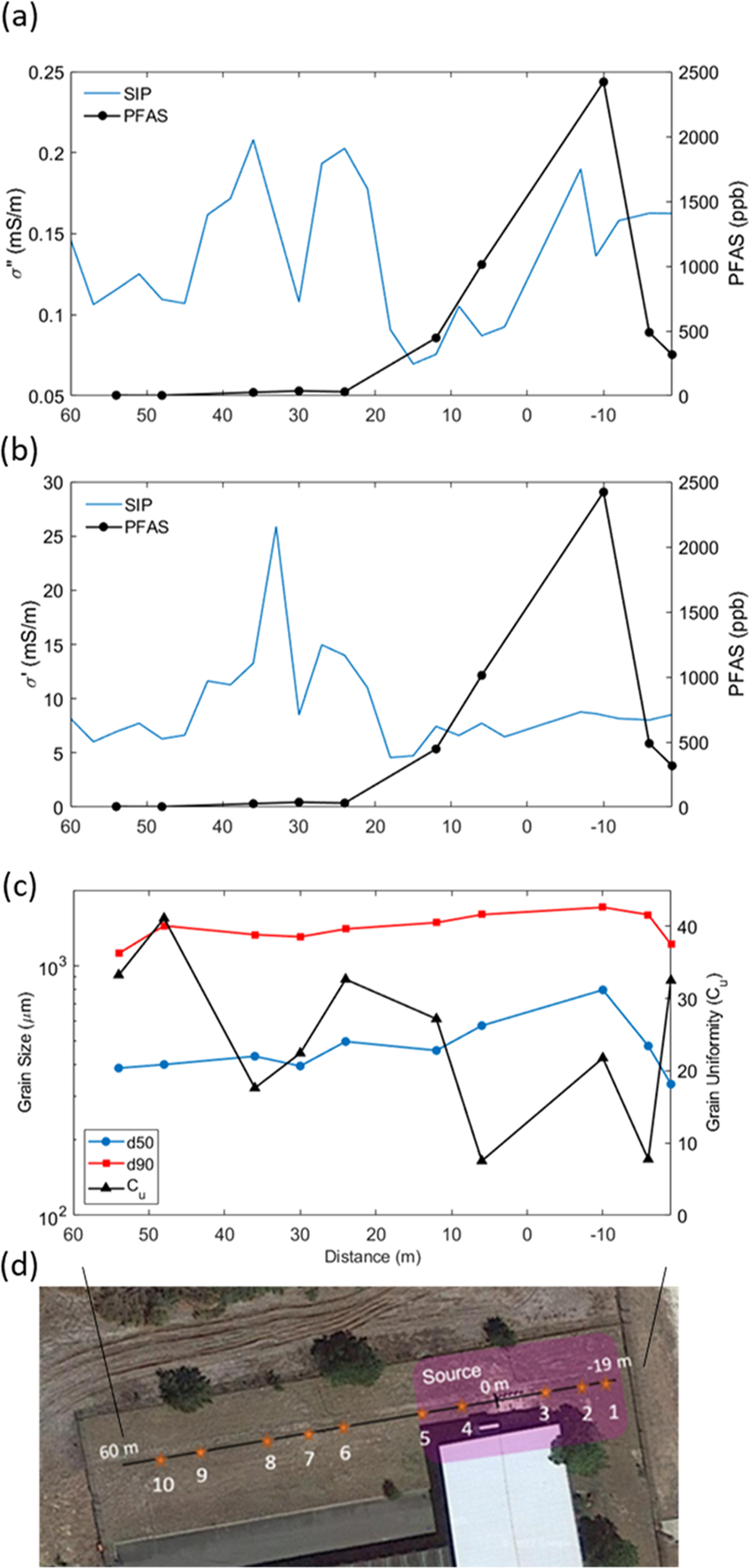
Profiles of imaginary (a) and real (b) conductivity from field-scale SIP measurements compared with PFAS concentrations from ten samples taken on a transect crossing the source zone at the study site (d). Variation in soil texture properties is shown in part (c). The equivalent results for the laboratory SIP measurements are shown in Fig. S1. (d50 = grain size for which 50% of the sample is finer by mass; d10 = grain size for which 10% of the sample is finer by mass; Cu = soil uniformity coefficient d60/d10).

Profiles of field-measured real and imaginary conductivity (at a frequency of 1 Hz) variations relative to total soil PFAS concentrations are shown in Fig. 3a–b. The plotted values are the averages of the five SIP measurements acquired over the depth range 0.18–0.4 m. The $\sigma^{\prime \prime }$ profile representing polarization (Fig. 3a) varies over a range typical for partially saturated soils devoid of electron-conducting minerals (between 0.07–0.21 mS/m). Two regions of high $\sigma^{\prime \prime }$ are observed, firstly between 20–45 m and second between −19 and 10 m along the profile. The latter region corresponds to the high PFAS concentrations in the source zone. The shape of the $\sigma^{\prime \prime }$ response along the transect varies little with frequency below 10 Hz. However, variations become significant in the 100–1000 Hz range, where instrumentation errors result in field-scale measurement uncertainty.

The corresponding $\sigma^{\prime }$ profile varies from 5–25 mS/m (Fig. 3b), which is typical of partially saturated soils. The shape of the $\sigma^{\prime }$ profile does not significantly vary with frequency. The profile shows one region of high $\sigma^{\prime }$ between 20–45 m, being consistent with the elevated $\sigma^{\prime \prime }$ profile at the same location. Unlike $\sigma^{\prime \prime },\;\sigma^{\prime }$ does not show any significant increase near the source zone between −19 and 10 m along the profile. Plots of soil texture parameters obtained from the analysis of the ten soil samples along the line indicate that the region of high $\sigma^{\prime \prime }$ and $\sigma^{\prime }$ between 20–45 m on the profile is likely attributable to a significant change in soil texture and its influence on surface conduction, with finer-grained (higher surface area), and less well-sorted soils further from the source zone (Fig. 3c).

The variations in $\sigma^{\prime }$ and $\sigma^{\prime \prime }$ determined from the saturated triplicate laboratory columns (Fig. S1) prepared from each of the ten soil samples obtained along the profile are consistent with the field measurements. However, both $\sigma^{\prime }$ and $\sigma^{\prime \prime }$ are consistently higher in the laboratory column experiments (by approximately an order of magnitude for $\sigma^{\prime }$ and a factor of six for $\sigma^{\prime \prime }$). This offset results from the differences in saturation state between the lab- and field-measured samples. Whereas the lab samples were prepared in a fully saturated state to avoid discrepancies in the measurements due to variations in saturation between samples, field measurements were conducted in situ under unsaturated conditions. Although moisture content for all field SIP measurement locations is unavailable, the average moisture content of the ten laboratory samples was 9.4% with a standard deviation of 1.63%. The $\sigma^{\prime }$ typically exhibits a power-law dependence on saturation (S)(S=θ/∅, where ∅ is the porosity and θ is the moisture content) with a saturation exponent (n) between 1.3–1.5 for typical soils [24]. The $\sigma^{\prime \prime }$ dependence on *S*is characterized by a smaller saturation exponent (p). In unconsolidated sediments, the approximation p=n-1 holds quite well [24].

**Fig. 4.**
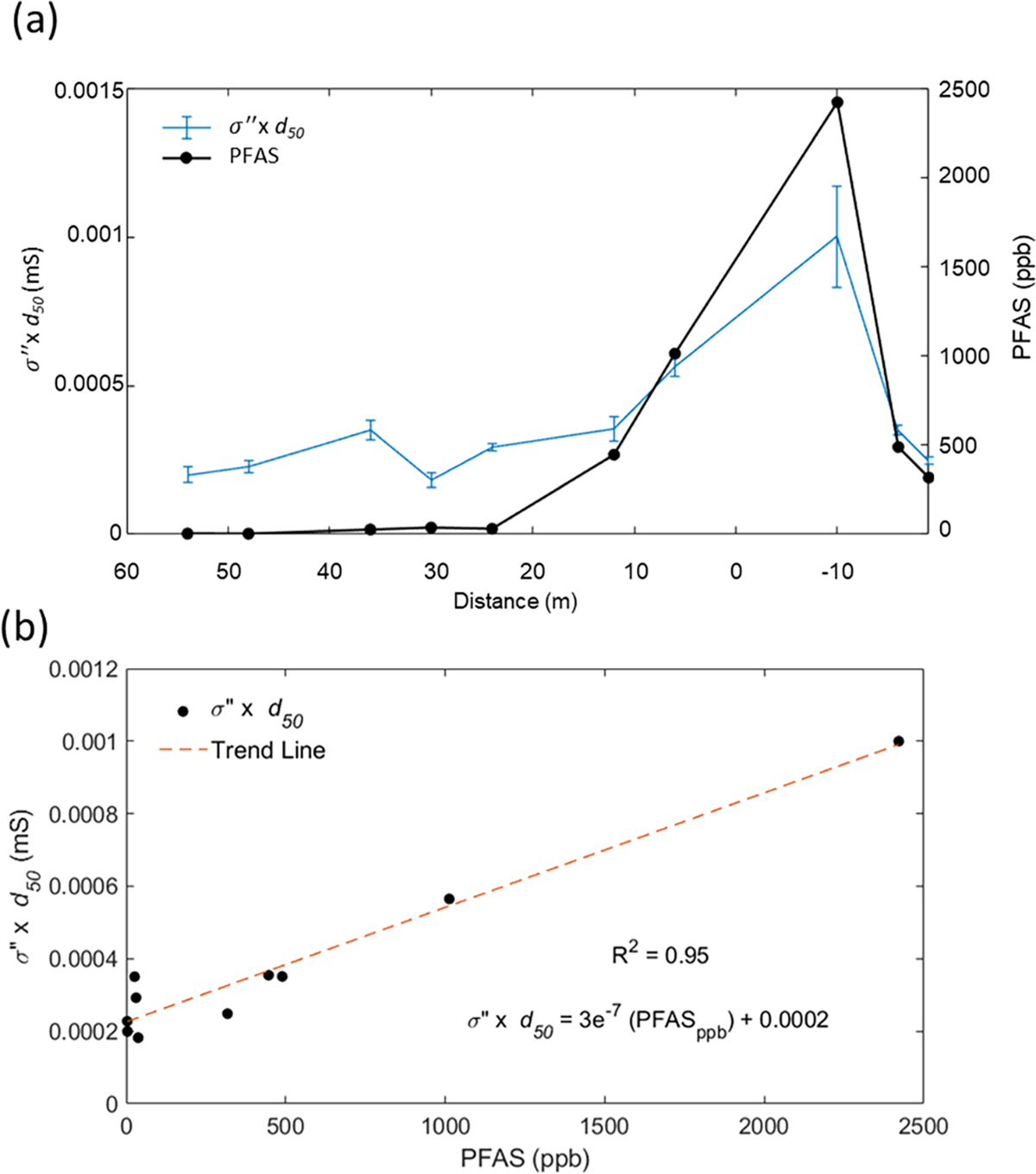
(a) Profile of laboratory-measured $\sigma^{\prime \prime }\times d_{50}$ from SIP measurements compared with PFAS concentrations from ten samples taken on a transect crossing the source zone at the study site (b). The linear relationship between laboratory-measured $\sigma^{\prime \prime }\times d_{50}$ and the measured total soil PFAS concentration (*p* value based t-test statistic = 0.037) (d50 = grain size for which 50% of the sample is finer than by mass).

The results presented in Fig. 3 and Fig. S2 highlight the limitations of SIP measurements for AFFF source zone characterization due to the strong dependence of the measurements on soil texture. Eqs. 5 and 7 emphasize that variations in soil texture ($S_{por},\;d_{0}$) must be removed from $\sigma^{\prime \prime }$ to reflect variations in the specific polarizability ($c_{p}$) resulting from AFFF interactions with the mineral surface. Although we do not have the information to calculate $c_{p}$ from Eq. 5, Eq. 7 states that the term $\sigma^{\prime \prime }\times d_{50}$ will be proportional to $c_{p}$ when variations in the formation factor are small (likely at this site since soil lithology does not vary dramatically along the line). Fig. 4 shows the correlation between $\sigma^{\prime \prime }\times d_{50}$ and PFAS concentration as (a) variations along the transect, and (b) as a cross-plot of $\sigma^{\prime \prime }\times d_{50}$ versus PFAS concentration. The calculation is based on the laboratory-measured $\sigma^{\prime \prime }$ (Fig. S1), as both SIP and grain size measurements are based on the laboratory-scale soil sample. The result is encouraging, with this proxy of $c_{p}$ tracking closely the total soil PFAS concentration.

**Fig. 5.**
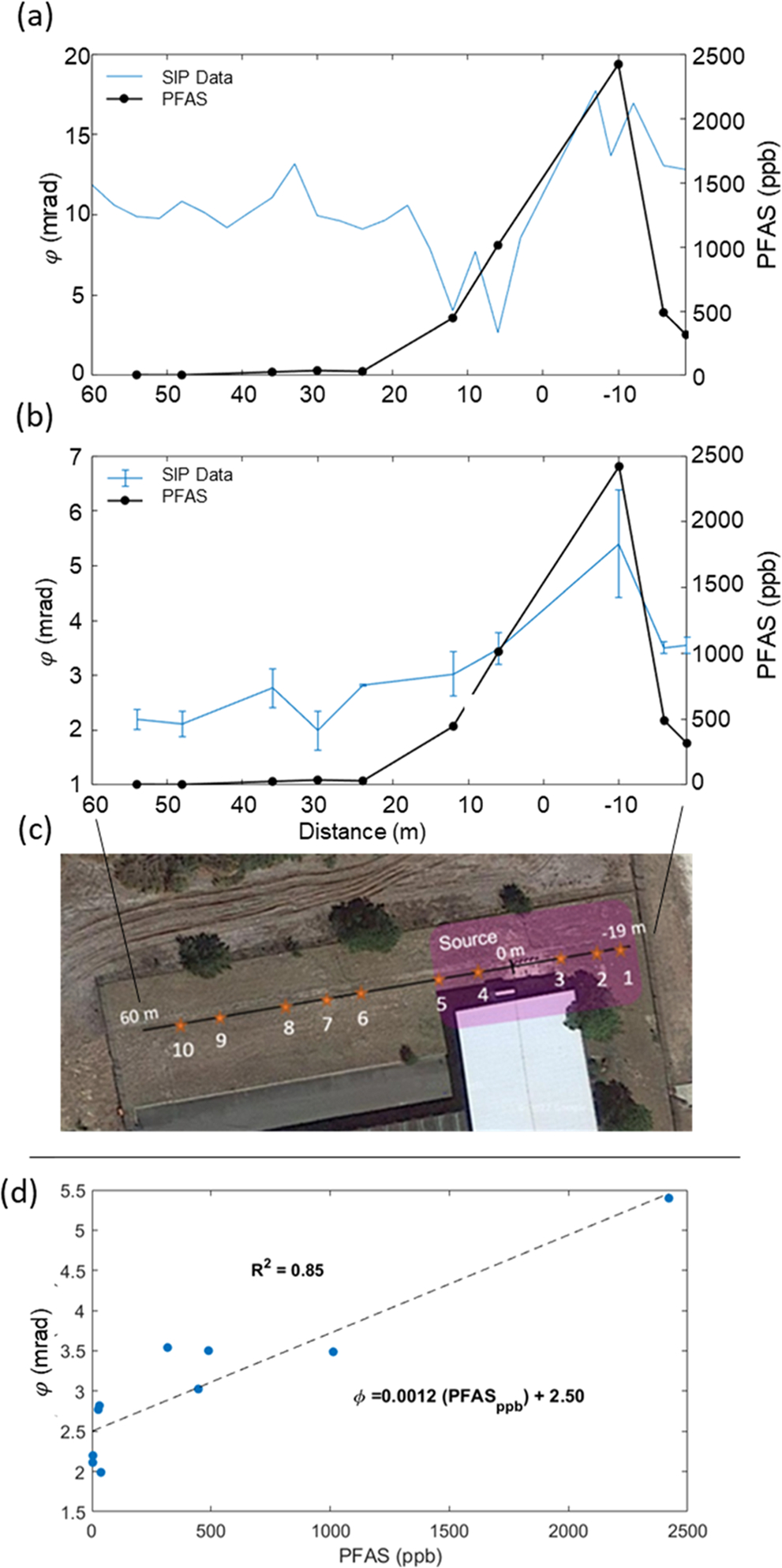
Profiles of field-measured (a) and laboratory-measured (b) phase from SIP measurements compared with PFAS concentrations from ten samples taken on a transect crossing the source zone at the study site (c). The linear relationship between laboratory-measured φ and the measured total soil PFAS concentration recorded is shown in (d) (*p*-value based t-test statistic = 0.038).

The above analysis indicates that information on grain size is needed to sense variations in AFFF contamination using SIP. However, recognizing that the real part of the surface conductivity $\sigma_{surf}^{\prime }$ is not sufficiently sensitive to AFFF contamination and will only sense lithological variation, the phase (the ratio of polarization strength ($\sigma^{\prime \prime }$) to conduction strength ($\sigma^{\prime }$), Eq. 1) potentially offers another way to reduce the influence of soil lithological variability on the CR response when surface conductivity changes dominate $\sigma^{\prime }$. The profile for the field-measured phase (again at 1 Hz) is shown in Fig. 5a. The φ varies from 3–17 mrad, being typical for partially saturated soils devoid of electron-conducting minerals. The φ is elevated for the five SIP measurement points located closest to the highest recorded PFAS concentration at −7 m on the profile. However, the lowest φ value along the profile is recorded at 9 m on the profile, coincidental with the second highest PFAS concentration recorded (1012 ppb). Away from the source zone, between 15–60 m on the profile, φ shows less variability, fluctuating between 9.5–13 mrad. Unlike the $\sigma^{\prime }$ and $\sigma^{\prime \prime }$ profiles, φ is not elevated in the region between 20–45 m that is attributed to the presence of finer-grained sediments.

Fig. 5b compares the φ for the SIP measurements on the saturated triplicate laboratory columns for each field sample (10 locations total) to the corresponding PFAS measurements on the portions of the field samples sent to the laboratory. Overall, φ is lower for the lab-measured samples relative to the field measurements of φ. For example, between 25–60 m on the profile, away from the source zone, lab-measured φ is between 2–3 mrad, whereas it is between 9.5–13 mrad in the field measurements. Considering the dependencies of $\sigma^{\prime }$ and $\sigma^{\prime \prime }$ on *S* as discussed above, Eq. 1 predicts that φ will be higher in the field measurements relative to the laboratory measurements due to the undersaturated state of the soils.

Similar to the field measured φ profile, lab-measured φ is highest over the PFAS source zone and tracks well the variations in PFAS along the profile based on the ten soil sampling locations. Also consistent with the field-measured φ, there is no increase in the lab-measured φ between 20–45 m along the line where finer-grained soils are located (Fig. 3c). The lab-measured φ does not show the anomalous decrease between 5–15 m on the profile that exists on the field-measured φ profile despite PFAS concentrations increasing relative to locations further from the source zone. Unlike the field-measured φ profile, the lab-measured φ values are significantly linearly correlated with the PFAS concentration, with a coefficient of determination (R^2^) equal to 0.85 (Fig. 3d). Nonlinear regression (using power law, logarithmic and exponential functions) did not result in a significant reduction in the residuals of this relationship.

### 5.3. SIP measurements as a function of frequency

**Fig. 6.**
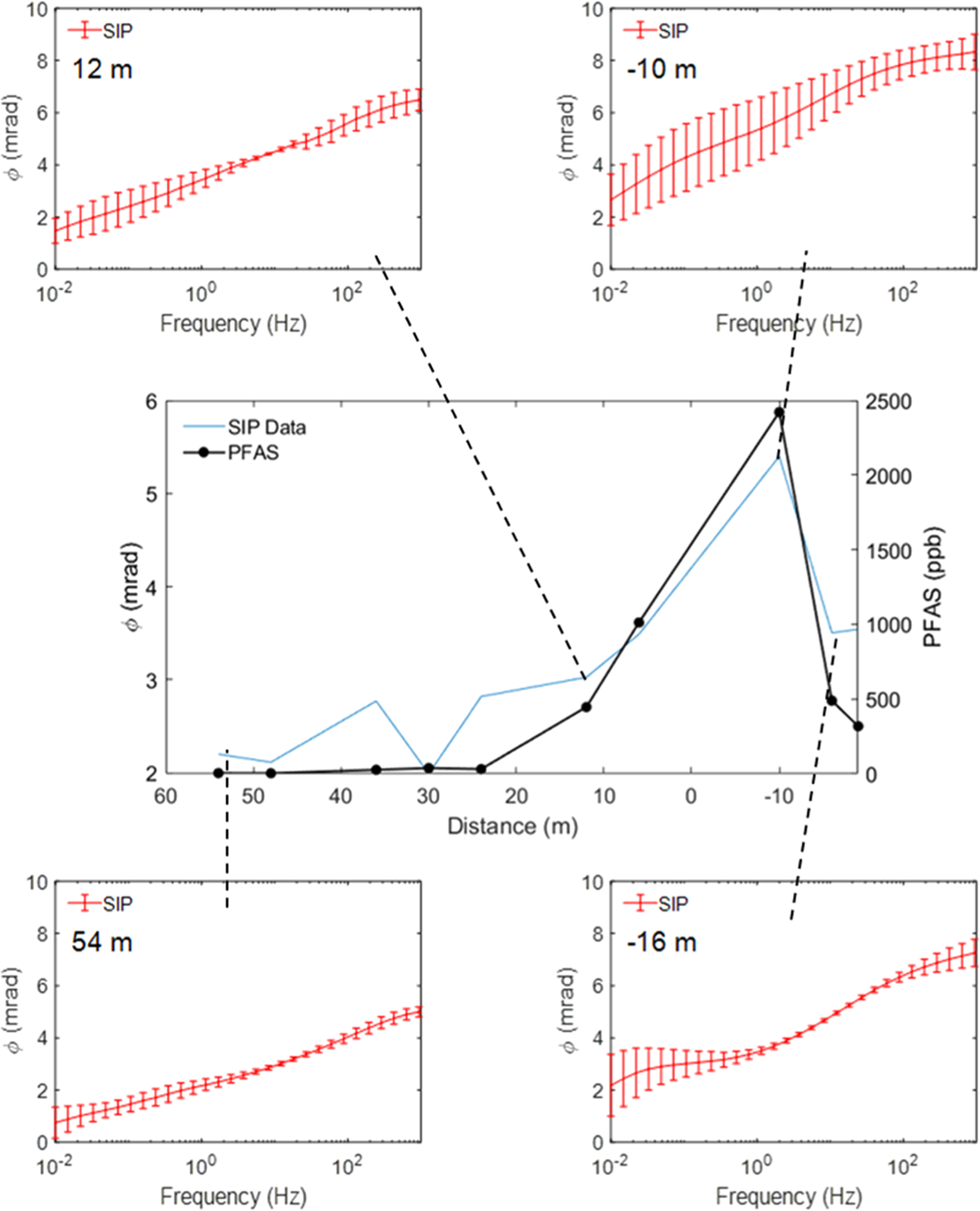
Selected laboratory-measured phase spectra for four points along the profile. Measurements at −10 m (top right) and −16 m (bottom right) are in the inferred source zone, the measurement at 12 m (top left) is on the edge of the inferred source zone, and the measurement at 54 m (bottom left) is far from the inferred source zone. Error bars represent standard deviations calculated from triplicate columns. The equivalent results for the field-measured phase spectra are shown in Fig. S2.

Fig. 6 shows selected laboratory-measured φ spectra (0.01–1000 Hz) for four of the ten samples acquired along the transect. Two locations in the source zone (−16 m and −10 m) are compared, with one location towards the edge of the source zone (12 m) and one location outside of the source zone (54 m). The region between 20–45 m along the profile where the soils are significantly finer is excluded to assess better the possibility of variations in the spectra being attributed to the presence of PFAS rather than to changes in texture. All spectra show a gradual increase in φ with frequency, being typical for unconsolidated soils and sediments [25]. Interestingly, the curvature of the spectra for the samples from the source zone (−16 m and −10 m) provides subtle evidence of the superposition of a low-frequency dispersion (φ peak around 0.1 Hz) and a higher frequency dispersion (φ peak around 1000 Hz) that are suppressed in the samples away from the source zone (12 m and 54 m). Fig. S2 shows the field-measured spectra (0.1–100 Hz) at the same locations. The shapes of the spectra are generally consistent between the lab- and field- measurements, although the subtle curvature of the spectra captured in the lab data is not noticeable in the field data. This similarity in the shape of the spectra foremost reflects the similarity in texture between the soil sample locations.

### 5.4. SIP response to a methanol wash procedure

**Fig. 7.**
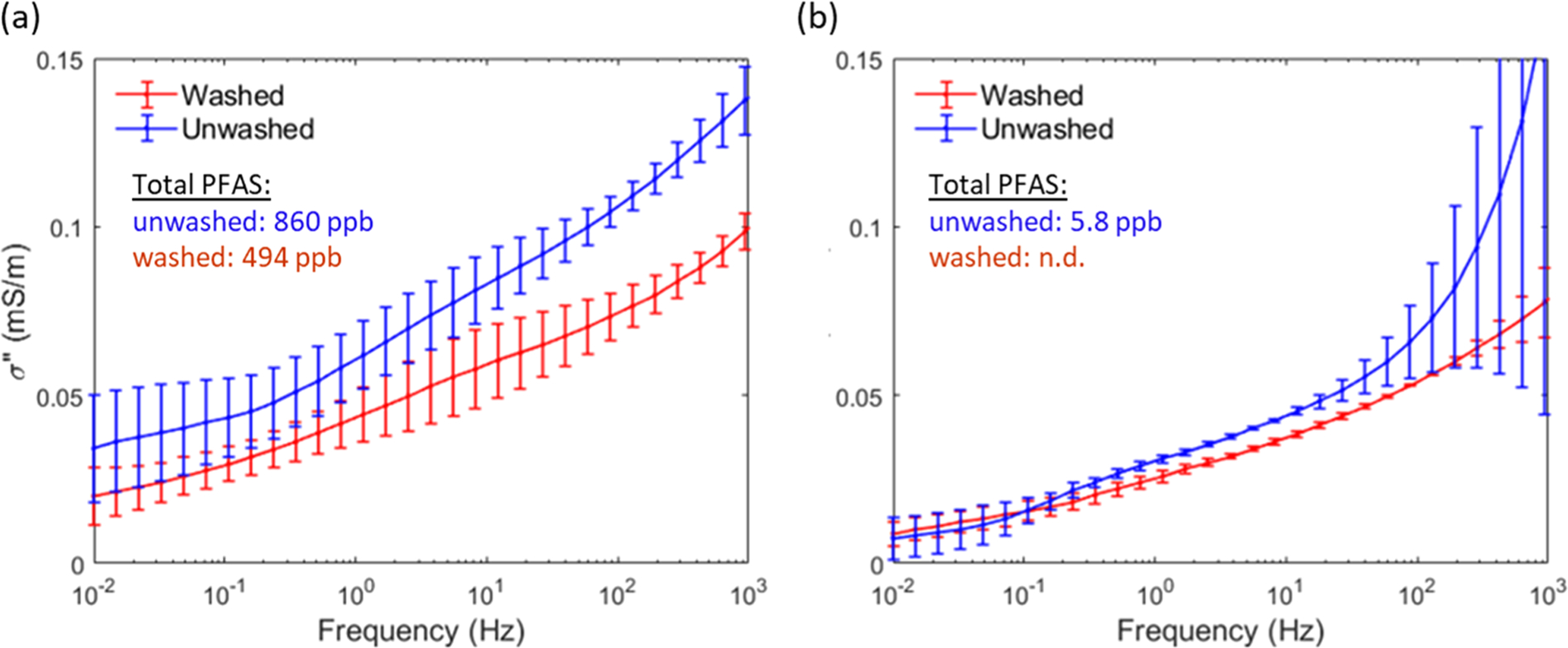
Imaginary conductivity spectra before (unwashed) and after (washed) a methanol wash procedure designed to remove soil-sorbed PFAS (a) contaminated sample M from the source zone (b) control sample MC far from the source zone. Fig. 2 shows the sample locations.

As soil texture does not change between the unwashed and the washed sample, the imaginary conductivity is plotted. Fig. 7 shows the laboratory measured $\sigma^{\prime \prime }$ response over the frequency range 0.01 – 10,000 Hz for the PFAS-contaminated soil from the source zone (sample M, Fig. 7a) compared to the control soil sample taken far from the source zone (sample MC, Fig. 7b). The washing procedure reduced the total PFAS concentration for the contaminated soil from 860 ppb to 494 ppb. For this soil, a significant (i.e., greater than the size of the error bars based on triplicate columns), approximately 57%, decrease in $\sigma^{\prime \prime }$ across the frequency range 1–1000 Hz, is observed. In contrast, the effect of the methanol wash procedure on the imaginary conductivity of the uncontaminated sample MC (Fig. 7b) is much smaller. For reference, $\sigma^{\prime }$ variations between the washed and unwashed contaminated samples were a maximum of 8% across the entire frequency range, and these differences fell within the error bars of the triplicate columns. In the case of the uncontaminated sample, $\sigma^{\prime }$ variations between the washed and unwashed samples are greater than the error bars, with a maximum 30% increase in $\sigma^{\prime }$ for the washed sample relative to the unwashed sample. This increase most likely reflects differences in the packing between samples and is unrelated to the presence of PFAS.

## 6. Discussion

This study investigated the hypothesis that variations in the concentrations of soil PFAS and/or non-fluorinated HCS could be detectable with the SIP electrical geophysical method. Findings from this study, involving SIP measurements across an AFFF source zone, lend support to this hypothesis. When $\sigma^{\prime \prime }$ is corrected for textural variations to provide a proxy for the specific polarizability ($\sigma^{\prime \prime }\times d_{50}$), a strong correlation with total PFAS concentration exists (Fig. 4). This indicates that spatial variations in soil texture (e.g., grain size distribution) would need to be recorded in addition to SIP, thereby limiting SIP as a rapid, non-invasive tool for AFFF source zone characterization. However, the phase of the complex conductivity from the SIP measurement (Eq. 1) provides another way to approximately account for the effects of varying soil texture on the SIP response. Using φ assumes that the real conductivity is not sensitive to the changes in the EDL chemistry caused by the AFFF interactions and that dividing the imaginary conductivity by the real conductivity removes the effect of surface conductivity changes due to varying soil texture. This approach assumes that the measured real conductivity is dominated by the surface conductivity term, as appears to be the case at this site. The φ measured in the laboratory on ten soil samples spanning the transect tracks the total measured PFAS concentrations determined for these samples. Despite the limited sample size, a statistically significant correlation between φ and total PFAS concentrations exists (Fig. 5d). Secondly, in situ measurements of φ from the field SIP measurements are elevated in the vicinity of the highest measured PFAS concentrations on the transect (Fig. 5a). However, a statistically significant correlation between in situ, field-measured φ and total PFAS measured on the soil samples was not found.

Laboratory measurements on contaminated and uncontaminated soil samples subjected to a methanol wash procedure support the hypothesis. A significant reduction in the imaginary conductivity over the frequency range 1–1000 Hz is associated with the loss of ~57% of the total soil PFAS following the wash of the contaminated soil. In contrast, there is no significant reduction in the SIP response for the control soil (Fig. 6). This SIP signal exists for a difference in soil sorbed total PFAS concentration of only 366 ppb between washed and unwashed soils. Variations in soil-sorbed total PFAS concentration are likely to be much greater for other source zones. For example, along the transect at this study site, PFAS concentrations in soils varied between 3 and 2420 ppb. In contrast, soil-sorbed PFAS concentrations as high as 70,000 ppb have been recorded on soils taken from other AFFF source zones [26].

The deployment of the SIP technology for rapid characterization of AFFF source zones will require statistical evidence that field-measured SIP data are significantly correlated with soil PFAS concentrations. This study fell short of meeting this requirement. Although the field-measured φ profile shows some clear correspondence with the variation in total PFAS determined for the samples, a statistically significant correlation was not established. This does not necessarily reflect a limitation of the SIP technology itself. It is plausible that this results from the fact that the sampled volume of soil is substantially different (and larger) for the field measurement relative to the field sample acquired for PFAS analysis. In contrast, the laboratory SIP measurements were performed on the same sample material sent off for PFAS analysis. One encouraging finding from the field measurements is that the increase in φ around the point of maximum PFAS contamination relative to the region away from the source zone is approximately the same, about 6 mrad, for both the field-measured φ and the lab-measured φ profile (Fig. 5). As previously discussed, the overall larger φ values recorded in the field relative to in the laboratory result from the differences in the saturation state between field and laboratory conditions. It is also encouraging that the frequency dependence of the field-measured φ spectra is generally consistent with the laboratory-measured φ spectra, indicating that reliable in situ field-scale SIP measurements of shallow, AFFF-contaminated soils is possible. However, any dependency of the shape of the spectra on AFFF contamination appears to be small, and unlikely to be resolvable in the field measurements.

A major limitation of any geophysical technology is that the results are often non-unique, given that geophysical properties are affected by multiple physical and chemical properties of the subsurface. A fundamental premise of this study is that any SIP response to soil PFAS and persistent non-fluorinated HCS concentrations would only be detectable in the polarization response ($\sigma^{\prime \prime }$) but not in the conduction response of the soil ($\sigma^{\prime }$). Both $\sigma^{\prime }$ and $\sigma^{\prime \prime }$ are strongly influenced by variations in soil texture properties, raising the concern that soil heterogeneity would mask any soil PFAS/HCS response. Our results indicate that this will not be the case. On the studied transect, soil textural variability (Fig. 3c) results in a region of high $\sigma^{\prime \prime }$ between 20–45 m that has a corresponding increase in $\sigma^{\prime }$, being diagnostic of a soil texture response (Fig. 3 and Fig. S1). In contrast, the rise in $\sigma^{\prime \prime }$ over the source zone is not associated with any increase in $\sigma^{\prime }$, being diagnostic of a change in the EDL surface chemistry (modifying the specific capacitance, $c_{p}$, Eq. 5) as a result of sorption of AFFF constituents that cannot be sensed with $\sigma^{\prime }$. The measured φ, being the ratio of $\sigma^{\prime \prime }$ to $\sigma^{\prime }$, is a convenient parameter to plot as, in this case, this ‘normalized’ (by conduction) polarization term does not sense the soil texture change along the transect and exclusively identifies an anomalously high polarization region associated with the source zone.

These findings encourage the development of SIP as a rapid, inexpensive sensor for mapping variations in soil PFAS concentrations across AFFF source zones. Although the method does not directly quantify soil PFAS concentrations, it has the potential to guide soil sampling, or more quantitative/specialized field sensors that may be developed in the future towards hotspots of AFFF contamination within source zones. However, more work is needed to evaluate whether sufficient signals will exist to detect variations in contaminant concentration at sites with more complex textural variability. Although soil texture varied on the investigated transect, other AFFF source zones may exhibit much more substantial lithological variations, e.g. transitions from sands to silts to clays. Furthermore, additional work is needed to better constrain the degree to which soil moisture content variations across a source zone might mask the SIP response to contamination.

## 7. Conclusions

Our findings present first evidence that the SIP geophysical technology is potentially deployable as a non-invasive field-scale screening technology for evaluating the extent of AFFF source zones. The technology could be used to design efficient soil sampling strategies that would improve AFFF source zone characterization and the assessment of total soil PFAS loads serving as long-term sources of groundwater contamination. Specifically, the technology might be used to rapidly delineate hot spots of soil PFAS contamination to be prioritized for sampling and analysis, thereby reducing the number of samples and the overall cost of delineating the impacted area compared to grid sampling. However, further field-scale experiments with the technology are needed to establish significant relationships between field-measured SIP data and soil PFAS concentrations. Ideally, these experiments would be performed over multiple, well-characterized AFFF source zones with differences in soil texture and mineralogical composition. Basic research on the SIP dependence on sorption of specific cationic or zwitterionic PFAS compounds, in addition to persistent non-fluorinated HCS, could improve understanding of the quantitative link between SIP and PFAS concentrations. However, the value of the technology is not in its potential to estimate soil PFAS concentrations but simply to rapidly screen a site for informed and effective implementation of soil sampling, followed by quantitative PFAS analysis methods.

## Supplementary Material

SI

## Data Availability

All data and supporting information relating to this work will be made available via the Hydroshare online collaboration environment for sharing data models and code.

## Environmental implication

The release of aqueous film-forming foam (AFFF) into the environment is an issue for water resources management that continues to grow in importance worldwide. In particular, using AFFF as a fire suppressant has a high potential for contaminating water resources. The poly- and per-fluoroalkyl substances (PFASs) associated with AF releases persist in the soil and serve as a long-term source of contamination to underlying groundwater. This paper reports promising, first field-scale results using spectral induced polarization, a non-invasive geophysical technique, for rapid and efficient characterization of AFFF source zones.

## HIGHLIGHTS

- Non-invasive spectral induced polarization (SIP) sensitive to soil PFAS concentrations.
- Specific polarizability linearly correlated with total soil PFAS concentrations (3– 2421 ppb).
- Phase (polarization/conduction) also linearly correlated with total soil PFAS concentration.
- Imaginary conductivity decreased when PFAS removed via methanol wash.
