# Supplementary material for: Spectral induced polarization (SIP) measurements across a PFAS-contaminated source zone
Source: J Hazard Mater. Author manuscript; Available in PMC 2025 Dec 5. (PMC11608158; doi:10.1016/j.jhazmat.2024.135829)
Supplement: SI [file NIHMS2032136-supplement-SI.docx]

# Supplemental material

Table S1: Target analytes measured using SGS AXYS MLA-110, which has been released by the U.S. Environmental Protection Agency (EPA) as EPA Method 1633. Total soil PFAS concentrations reported in this paper represent the sum of all analytes listed in this table.

| \| **Perfluoroalkyl carboxylates**  Perfluorobutanoic acid (PFBA, Perfluorobutanoate)  Perfluoropentanoic acid (PFPeA, Perfluoropentanoate)  Perfluorohexanoic acid (PFHxA, Perfluorohexanoate)  Perfluoroheptanoic acid (PFHpA, Perfluoroheptanoate)  Perfluorooctanoic acid (PFOA, Perfluorooctanoate)  Perfluorononanoic acid (PFNA, Perfluorononanoate)  Perfluorodecanoic acid (PFDA, Perfluorodecanoate)  Perfluoroundecanoic acid (PFUnA, Perfluoroundecanoate)  Perfluorododecanoic acid (PFDoA, Perfluorododecanoate)  Perfluorotridecanoic acid (PFTrDA, Perfluorotridecanoate)  Perfluorotetradecanoic acid (PFTeDA, Perfluorotetradecanoate) \| \| --- \| \| **Perfluoroalkyl sulfonates**  Perfluorobutanesulfonic acid (PFBS, Perfluorobutanesulfonate)  Perfluoropentanesulfonic acid (PFPeS, Perfluoropentanesulfonate)  Perfluorohexanesulfonic acid (PFHxS, Perfluorohexanesulfonate)  Perfluoroheptanesulfonic acid (PFHpS, Perfluoroheptanesulfonate)  Perfluorooctanesulfonic acid (PFOS, Perfluorooctanesulfonate)  Perfluorononanesulfonic acid (PFNS, Perfluorononanesulfonate)  Perfluorodecanesulfonic acid (PFDS, Perfluorodecanesulfonate)  Perfluorododecanesulfonic acid (PFDoS, Perfluorododecanesulfonate) \| \| **Fluorotelomer sulfonates**  1H, 1H, 2H, 2H-perfluorohexane sulfonic acid (4:2 FTS, 1H, 1H, 2H, 2H-perfluorohexane sulfonate)  1H, 1H, 2H, 2H-perfluorooctane sulfonic acid (6:2 FTS, 1H, 1H, 2H, 2H-perfluorooctane sulfonate)  1H, 1H, 2H, 2H-perfluorodecane sulfonic acid (8:2 FTS, 1H, 1H, 2H, 2H-perfluorodecane sulfonate) \| \| **Fluorotelomer carboxylates**  2H, 2H, 3H, 3H-perfluorohexanoic acid (3:3 FTCA, 2H, 2H, 3H, 3H-perfluorohexanoate)  2H, 2H, 3H, 3H-perfluorooctanoic acid (5:3 FTCA, 2H, 2H, 3H, 3H-perfluorooctanoate)  2H, 2H, 3H, 3H-perfluorodecanoic acid (7:3 FTCA, 2H, 2H, 3H, 3H-perfluorodecanoate) \| \| **Perfluorooctane sulfonamides**  Perfluorooctanesulfonamide (PFOSA, FOSA)  N-Methylperfluorooctanesulfonamide (NMeFOSA)  N-Ethylperfluorooctanesulfonamide (NEtFOSA) \| \| **Perfluorooctane sulfonamidoacetic acids**  N-Methylperfluoro-1-octanesulfonamidoacetic acid (NMeFOSAA, N-Methylperfluorooctanesulfonamidoacetate)  N-Ethylperfluoro-1-octanesulfonamidoacetic acid (NEtFOSAA, N-Ethylperfluorooctanesulfonamidoacetate) \| \| **Perfluorooctane sulfonamidoethanols**  N-Methylperfluorooctanesulfonamidoethanol (NMeFOSE)  N-Ethylperfluorooctanesulfonamidoethanol (NEtFOSE) \| \| **Ether carboxylates**  Hexafluoropropylene oxide dimer acid (HFPO-DA, 2,3,3,3-Tetrafluoro-2-(1,1,2,2,3,3,3-heptafluoropropoxy)propionoate)  4,8-Dioxa-3H-perfluorononanoic acid (ADONA, DONA, 4,8-Dioxa-3H-perfluorononanoate) Nonafluoro-3,6-dioxaheptanoate (NFDHA, nonafluoro-3,6-dioxaheptanoic acid)  Perfluoro-3-methoxypropanoate (PFMPA, Perfluoro-3-methoxypropanoic acid)  Perfluoro-4-methoxybutanoate (PFMBA, Perfluoro-4-methoxybutanoic acid) \| \| **Ether sulfonates**  9-chlorohexadecafluoro-3-oxanonane-1-sulfonic acid (9Cl-PF3ONS, 9-chlorohexadecafluoro-3-oxanonane-1-sulfonate)  11-chloroeicosafluoro-3-oxaundecane-1-sulfonic acid (11Cl-PF3OUdS, 11-chloroeicosafluoro-3-oxaundecane-1-sulfonate)  Perfluoro(2-ethoxyethane)sulfonic acid (PFEESA, Perfluoro(2-ethoxyethane)sulfonate \| |
| --- | --- | --- | --- | --- | --- | --- | --- | --- | --- |
|  |

|  |
| --- |
|  |

Table S2: Composition of the synthetic groundwater constructed to represent the study site.

| **Compound** | **Concentration (g/L)** |
| --- | --- |
| NaNO_3_ | 0.6075 g |
| MnSO_4_ – H_2_O | 0.001 g |
| Na_2_SO_4_ | 0.18 g |
| NaCl | 0.113 g |
| NaHCO_3_ | 0.04 g |


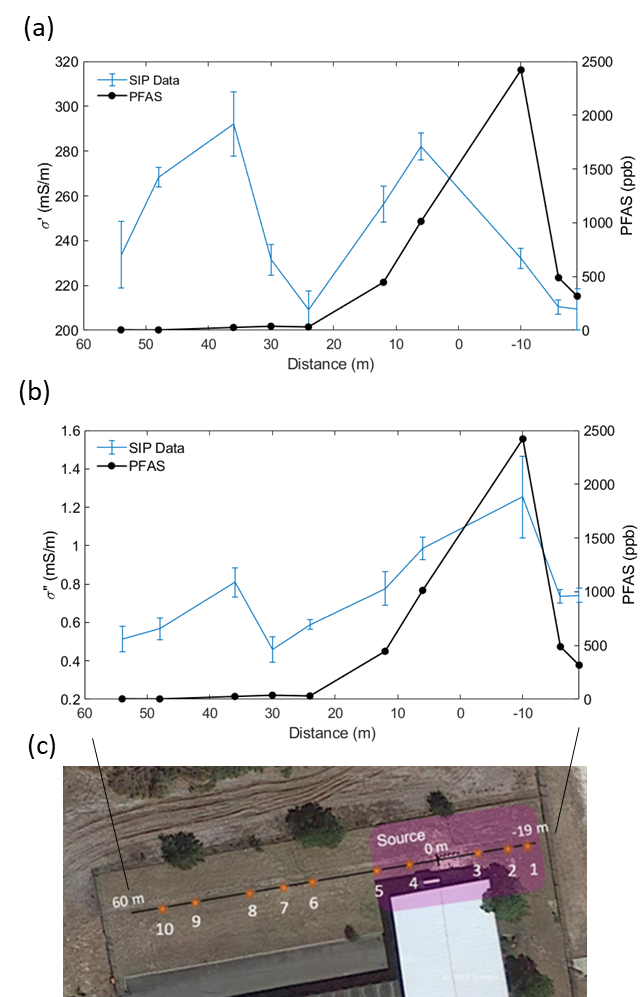

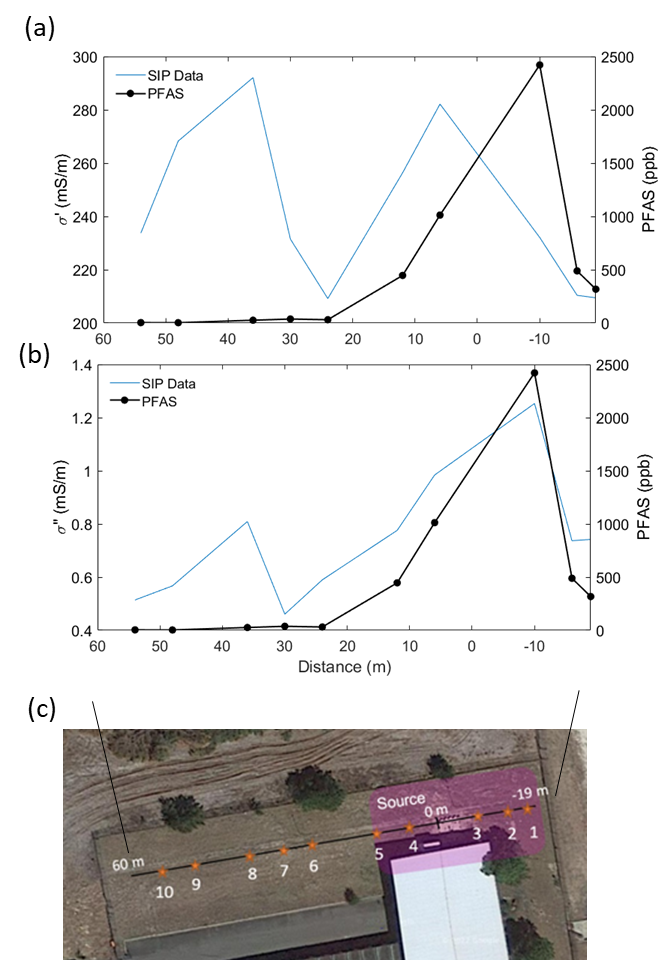


Figure S1: Profiles of real (a) and imaginary conductivity (b) from laboratory SIP measurements compared with PFAS concentrations from ten samples taken on a transect crossing the source zone at the study site (c). The equivalent results for the field SIP measurements are shown in Figure 3.


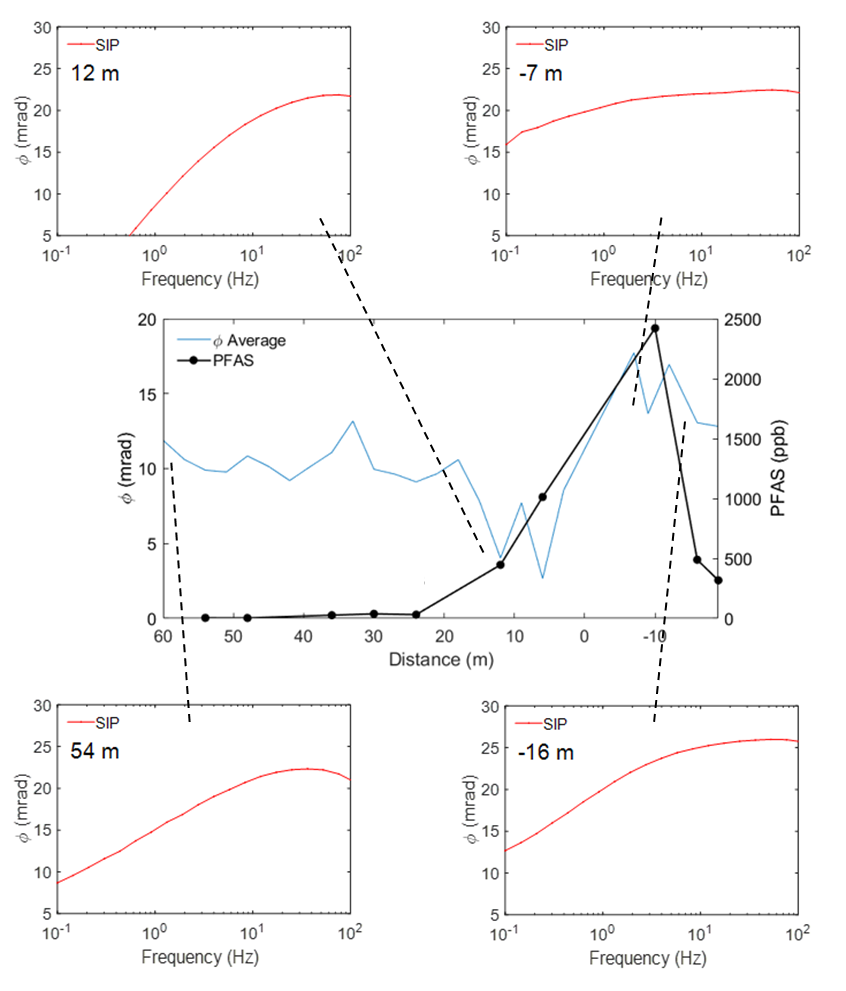


Figure S2: Selected field-measured phase spectra for four points along the profile at the study site. Measurements at -7 m (top right) and -16 m (bottom right) are in the inferred source zone, the measurement at 12 m (top left) is on the edge of the inferred source zone and the measurement at 54 m (bottom left) is far from the inferred source zone. The equivalent results for the laboratory-measured phase spectra are shown in Figure 5.
